# Effect of regional body composition changes on bone density remodeling after sleeve gastrectomy

**DOI:** 10.3389/fendo.2023.1238060

**Published:** 2023-09-11

**Authors:** Di Yang, Rongrong Xu, Yinfang Tu, Yunfeng Xiao, Hongwei Zhang, Weijie Liu, Pin Zhang, Haoyong Yu, Yuqian Bao, Ying Yang, Junfeng Han

**Affiliations:** ^1^ Department of Endocrinology and Metabolism, Shanghai Sixth People’s Hospital Affiliated to Shanghai Jiao Tong University School of Medicine, Shanghai Key Laboratory of Diabetes Mellitus, Shanghai, China; ^2^ Department of Radiology, Shanghai Sixth People’s Hospital Affiliated to Shanghai Jiao Tong University School of Medicine, Shanghai, China; ^3^ Department of General Surgery, Shanghai Sixth People’s Hospital Affiliated to Shanghai Jiao Tong University School of Medicine, Shanghai, China; ^4^ Department of Endocrinology and Metabolism, Tongji Hospital, School of Medicine, Tongji University, Shanghai, China

**Keywords:** sleeve gastrectomy, body composition, bone mineral density, bone turnover markers, obesity

## Abstract

**Background:**

Sleeve gastrectomy (SG) results in bone mineral density (BMD) loss and varying body composition parameters. However, the effects of body compositions on bone health are controversial. In order to accurately demonstrate their relationship and provide new insights into the causes of BMD loss after sleeve gastrectomy, this study is aimed to investigate the role of changes in body composition in BMD loss 12 months after SG.

**Methods:**

41 Chinese individuals with obesity (25 women and 16 men) who underwent SG were prospectively examined for at least 12 months. Measurements of anthropometrics, body composition, BMD and blood samples were collected.

**Results:**

For 12 months, the femoral neck (FN) BMD and total hip (TH) BMD decreased significantly compared with baseline in both sexes but not lumbar spine (LS) BMD. Greater TH BMD loss was observed in men than in women. For the first 6 months post-SG, the FN BMD loss was positively associated with the estimated fat free mass index (eFFMI) reduction in women (adjusted β = 0.77, P = 0.004) and positively associated with reduction of subcutaneous fat area (SFA) in men (r = 0.931, P = 0.007). For 12 months post-SG, the FN BMD loss was negatively associated with visceral fat area (VFA) reduction in women (adjusted β = -0.58, P = 0.027) and men (adjusted β = -0.68, P = 0.032). TH BMD loss was positively associated with waist circumference reduction in women (r = 0.448, P = 0.028).

**Conclusion:**

FN and TH BMD decrease after SG in both women and men. The changes in body compositions are associated with BMD loss at different time points and bone sites. Our data emphasize the limitation of simply taking the total weight loss (% TWL) as an influencing factor of bone mineral density and the necessity of delineating body composition in relevant studies.

## 1 Introduction

Sleeve gastrectomy (SG) is one of effective bariatric surgery (BS) types for achieving long-term weight loss and improving a wide range of obesity-related comorbidities (1). However, numerous studies have extensively documented the impact of SG on bone mass presented as bone mineral density (BMD) loss (2–4). Multiple mediators may be involved in the bone loss occurring after BS such as the rebuilding of the gastrointestinal anatomy, impairment of calcium, vitamin D absorption, and variation of the hormone spectrum (5, 6). Furthermore, the unloading of the skeleton combined with weight loss has been considered among the main factors (7). Besides, the literature is still controversial regarding the relationship between body composition and BMD. Some studies have found that fat mass and lean mass are beneficial for BMD (8, 9). However, other studies have reported that fat mass have opposite impact on BMD and the relationship varied with the degree of obesity (10). Most of the current studies focusing on the relationship between body composition and BMD are cross-sectional studies, and little is known about their relationships in bariatric surgery longitudinally. Therefore, the further study is warranted to investigate the relationship between changes in body composition and BMD in patients received SG.

Unlike Dual-Energy X-ray absorptiometry (DEXA) (11–13), which lacks the ability to accurately distinguish between various types of fat tissue such as visceral fat area (VFA) and subcutaneous fat area (SFA), magnetic resonance imaging (MRI) is considered the gold standard method for accurately measuring regional body composition without causing radiation damage (14). Cross-sectional imaging at the third lumbar vertebra level (L3) is widely used to assess the whole-body composition as per the National Institutes of Health (15). The measurement of VFA is used to define abdominal obesity in Chinese (16), while the psoas muscle area (PMA) provides an effective estimate for overall muscle mass (17). Among Chinese participants who underwent SG, the association between the MRI-derived changes in precisely distinguished regional body composition and BMD loss is unknown.

Therefore, the primary aim of this study was to identify whether changes in the body composition parameters could affect BMD loss within 12 months after SG. The secondary objective was to investigate the potential intrinsic effect of various body composition parameters on BMD loss at different sites longitudinally.

## 2 Materials and methods

### 2.1 Study design and participants

This is a *post hoc* analysis of a prospective bariatric surgery cohort conducted at the bariatric center of Shanghai Jiao Tong University school of medicine Affiliated Sixth People’s Hospital, China. We recruited patients with obesity (women aged < 45 years with regular menstrual cycles and men aged < 50 years) who underwent SG per Chinese guidelines criteria and completed the comprehensive medical examination preoperatively (18). The exclusion criteria were as follows: (1) bone diseases of different etiologies (primary hyperparathyroidism, Paget disease, clinical or laboratory evidence of hepatic or renal failure, previous BS, or excessive alcohol intake (> 3 U/d)); (2) use of medications affecting bone metabolism, including bisphosphonates or teriparatide (in the last year or for > 12 months), oral glucocorticoids, insulin, thiazolidinediones, calcium, and vitamin D supplementations; (3) specific endocrine diseases that could cause obesity, such as hypothyroidism, polycystic ovarian syndrome and pituitary tumor; (4) missing data on age, sex, body mass index (BMI), or laboratory investigations; (5) gestation or lactation or malignant disease; (6) missing the 12 month’s follow-up visit.

All patients were prospectively examined at baseline and 6, 12 months postoperatively. 50 μg of vitamin D3 and 600 mg of calcium were prescribed as an oral supplement in all patients postoperatively daily (19). All participants signed informed consent. This study has been registered in the Chinese Clinical Trial Registry (ChiCTR2000037647).

### 2.2 Sleeve gastrectomy procedures

Laparoscopic SG involved removing a major portion of the gastric greater curvature. A 32-36 French oral gastric tube was placed through the pylorus into the duodenal and left near the lesser curve. The dissection was started 2–6 cm from the pylorus and straight toward the cardiac notch angle of His over the 32-36 French bougie. The total fundus was removed with the antrum partially retained. Two metabolic surgeons performed the SG (20) operations for all patients using standardized laparoscopic techniques.

### 2.3 Anthropometric measurements

Demographic and health condition data, including age, sex, medical history, were recorded preoperatively. Anthropometric variables were measured at each visit. Details of recording the anthropometric measurements, including height (cm), weight (kg), BMI (kg/m^2^), waist circumference, systolic blood pressure, and diastolic blood pressure, have been described previously (19). The excess weight loss percent (%EWL) was calculated as follows: 100× (preoperative weight−current weight)/(preoperative weight−ideal weight); BMI of 24 kg/m^2^ was used to calculate the ideal weight (21). The percent of total weight loss (%TWL) was calculated as 100 × (initial weight - postoperative weight)/initial weight. The estimated body fat percent (%eBF) was calculated as follows: (1.20 × BMI) + (0.23 × age) − (10.8 × sex) − 5.4, with 1 used for men and 0 for women. The estimated fat-free mass index (eFFMI) was calculated as follows: BMI × (1− %eBF/100) (22).

### 2.4 Biochemical measurements

Biochemical variables were measured at each visit. A 12-h overnight fasting blood sample was collected for all participants to determine the bone metabolism, lipid profile, fasting plasma glucose (FPG), fasting insulin, glycated hemoglobin (HbA1c), total serum protein (TSP) and serum albumin (SA). Homeostatic Model Assessment for Insulin Resistance (HOMA-IR) was assessed as fasting insulin (mIU/L) × fasting glucose (mM)/22.5. HOMA-β was calculated as follows: (20 × insulin [mIU/mL])/(glucose [mmol/L]– 3.5), as previously described (23). Bone metabolism parameters, including parathyroid hormone (PTH), 25-hydroxy vitamin D (25[OH]D), procollagen type I amino-terminal propeptide (P1NP), N-terminal osteocalcin (N-MID), and C-terminal telopeptide of type 1 collagen (β-CTX) were measured using an automated Roche electrochemiluminescence system (Roche Diagnostics GmbH, Germany).

### 2.5 Bone mineral density measurements

Areal BMD (g/cm^2^) was measured using DEXA preoperatively and at 6 and 12 months postoperatively. BMD data of the lumbar spine (LS1–4), femoral neck (FN), and total hip (TH) were recorded. All DEXA scans were performed using Hologic QDR-2000 (Hologic Corporation, Waltham, MA, USA) by two experienced operators following standard manufacturer protocols, as described previously in detail (19). Daily measurement of the Hologic DEXA spine phantom was performed for quality control maintenance. The coefficient of variation for BMD was 0.8–1.0%. Prodigy encore software (version 6.70, standard-array mode; GE Healthcare) was used for data analysis.

### 2.6 Measurements of body composition

Body composition parameters, including VFA, SFA, and PMA, were measured using Achieva 3.0T MRI system (Philips Healthcare, Eindhoven, Netherlands). A medically trained technician analyzed a 10-mm slice at the L3 level with good contrast using sliceOmatic software (version 5.0; TomoVision, Magog, Canada). The software calculates the areas of different tissues expressed as cm^2^, as previously reported (24).

### 2.7 Statistical analysis

The Shapiro–Wilk test was used to evaluate the normality of all continuous variables. Variables with a normal distribution were presented as means ± standard deviations, while the skewed variables were expressed as medians (interquartile ranges). A paired-sample t-test or Wilcoxon signed-rank test was used to compare the changes in the clinical characteristics at baseline and 6 or 12 months after BS. GLMM (generalized linear mixed model) was used to evaluate time × sex interactions for participants undergoing SG. With adjustment for baseline values, the percent change from baseline to 6 months or 12 months for the parameters was calculated as follows: 100 × (initial measurement–postoperative measurement)/initial measurement. Pearson or Spearman analysis was used to assess the correlations between BMD and clinical variables including body composition parameters. Multiple stepwise regression analysis was used to estimate the influence of different body composition parameters on BMD at different sites. The results were presented as adjusted regression coefficient (β) values and adjusted coefficient of determination (R^2^). All analyses were 2-tailed with a significance level (alpha) of 0.05. All data analyses were performed using IBM Statistical Product and Service Solutions Statistics for Windows, version 26.0 (IBM Corp, Armonk, NY, USA). All graphs were made using Graphpad Prism 8.0 (GraphPad Software, San Diego, CA, USA). The *post hoc* power analysis was calculated using Power Analysis and Sample Size 2021 (NCSS, USA).

## 3 Results

### 3.1 Changes in metabolic characteristics after sleeve gastrectomy

A total of 41 participants (25 non-menopausal adult women and 16 men) were invited. The mean age was 31.62 ± 6.72 years with no significant difference between genders. The changes in metabolic indicators are shown in **Table 1**. As expected, most of the glucose–lipid metabolic parameters including total glycerides (TG), low-density lipoprotein cholesterol (LDL-c), FPG, HbA1c reduced significantly from baseline to 12 months postoperatively (P < 0.05). As for factors related to bone metabolism, 25[OH]D increased significantly in both genders from baseline to 6 months postoperatively, while calcium level showed no significant changes in both genders, although these patients took vitamin D3 and calcium postoperatively daily. Additionally, PTH decreased significantly at 12 months after surgery in women while significantly decreased 6 months after surgery in men.

**Table 1:** Metabolic characteristics of the participants at baseline and at 6 and 12 months after sleeve gastrectomy.

	Women					Men				
	Baseline	6 months	p-value	12 months	p-value	Baseline	6 months	p-value	12 months	p-value
Demographics										
Age, years	31.88 ± 6.49	–		–		31.08 ± 7.44	–		–	
Laboratory data										
TC, mM	4.79 ± 0.71	4.80 ± 0.96	0.987	4.40 ± 0.64	0.002	4.50 ± 1.22	4.32 ± 1.21	0.899	3.90 ± 0.73	0.09
TG, mM	1.64 ± 0.65	0.91 ± 0.28	<0.001	0.76 ± 0.23	<0.001	2.35 ± 1.49	0.98 ± 0.35	0.009	1.03 ± 0.57	0.007
HDL-C, mM	1.07 ± 0.2	1.16 ± 0.20	0.029	1.36 ± 0.26	<0.001	0.87 ± 0.15	1.31 ± 0.27	<0.001	1.27 ± 0.26	<0.001
LDL-C, mM	3.04 ± 0.72	2.95 ± 0.80	0.65	2.36(2.23,2.93)	0.003	2.80 ± 1.04	2.55 ± 0.86	0.799	2.18 ± 0.55	0.024
FPG, mM	5.43(4.87,7.72)	4.80 ± 0.63	<0.001	4.48 ± 0.38	<0.001	8.35 ± 2.67	4.82 ± 0.63	0.006	4.71 ± 0.80	<0.001
PBG, mM	8.32 ± 4.08	4.92(4.48,5.54)	0.004	4.83 ± 1.15	<0.001	12.03 ± 4.93	5.22 ± 0.81	0.009	6.12 ± 2.10	<0.001
HbA1c, %	5.65(5.2,8.3)	5.44 ± 0.57	<0.001	5.38 ± 0.46	0.031	8.34 ± 1.93	5.5 ± 0.41	<0.001	5.53 ± 0.39	<0.001
FINS, Mu/L	24.76 ± 15.35	8.54 ± 5.94	<0.001	6.88 ± 3.60	<0.001	26.39 ± 19.06	6.31(3.12,17.11)	0.017	6.22 ± 3.74	0.005
HOMA-IR	7.24 ± 7.57	1.86 ± 1.42	0.002	1.43 ± 0.81	<0.001	7.72(4.12,16.17)	2.28 ± 2.31	0.012	1.58 ± 1.17	0.003
HOMA-β	245.33 ± 126.61	109.93(81.15,190.16)	0.039	132.62 ± 44.94	0.003	140.97 ± 137.21	116.38(54.50,245.57)	0.889	103.27(68.53,174.16)	0.657
25(OH)D, mM	13.41 ± 3.08	21.21 ± 8.95	0.003	19.02 ± 10.06	0.026	14.54 ± 5.17	23.33 ± 8.31	0.005	16.76 ± 5.11	0.072
Calcium, mM	2.24 ± 0.07	2.30 ± 0.11	0.041	2.21 ± 0.1	0.128	2.30 ± 0.10	2.37 ± 0.1	0.167	2.28 ± 0.09	0.497
Phosphorus,mM	1.27 ± 0.16	1.37 ± 0.22	0.131	1.37 ± 0.13	0.015	1.31 ± 0.13	1.45 ± 0.14	0.427	1.45 ± 0.12	0.006
PTH, pg/ml	44.82 ± 11.83	41.58 ± 18.36	0.365	37.42 ± 15.69	0.046	37.68 ± 12.53	25.23 ± 6.64	0.013	41.58 ± 13.34	0.873
TSP, g/L	68.54 ± 4.97	67.00 ± 4.81	0.937	63.83 ± 3.79	0.002	69.00 ± 6.99	70.33 ± 4.39	0.683	67.18 ± 4.97	0.412
SA, g/L	43.67 ± 3.61	42.53 ± 4.24	0.639	39.93 ± 3.08	<0.001	46.00(43.00,50.00)	44.67 ± 3.32	0.484	43.08 ± 3.21	0.227

TC, total cholesterol; TG, total triglycerides; HDL-C, high-density lipoprotein cholesterol; LDL-C, low-density lipoprotein cholesterol; FPG, fasting plasma glucose; PBG, postprandial blood glucose; HbA1c, glycated hemoglobin; FINS, fasting insulin; HOMA-IR, homeostasis model assessment of insulin resistance; HOMA-β, homeostasis model assessment of β-cell function; 25(OH)D, serum 25-hydroxyvitamin D; PTH, parathyroid hormone; TSP, total serum protein; SA, serum albumin.

Data are presented as means ± standard deviations, or medians (Q25, Q75).

P values indicate statistical significance compared with baseline in each sex group.

### 3.2 Changes in body composition parameters after sleeve gastrectomy

The changes in body composition parameters are shown in **Table 2**. The mean BMI at baseline was 35.05 ± 5.06 kg/m^2^ with no significant difference between genders. At 12 months postoperatively, the mean reduction in the %TWL and %EWL was 29.13% and 90% in women, and 26.95% and 91% in men, respectively. In the first 6 months, significant decreases in VFA, SFA, PMA and eFFMI values were observed regardless of gender, while only female showed a continued decline for 12 months. Across sex, men demonstrated greater VFA reduction than women at 6 months. eFFMI in men declined more than in women at both 6 and 12 months. Sex-specific responses of SG for body composition parameters were shown in GLMMs (**Table 3**). The *post hoc* statistical power (%) of VFA, SFA, and eFFMI in paired-sample t-test was 99.82%, 100%, and 100% for women group; 98.62%, 99.95% and 100% for men group, respectively.

**Table 2:** Body composition parameters of the participants at baseline and at 6 and 12 months after sleeve gastrectomy.

	Women					Men				
	Baseline	6 months	p-value	12 months	p-value	Baseline	6 months	p-value	12 months	p-value
Height, cm	163.96 ± 6.23	–		–		174 ± 5.19	–		–	
Weight, kg	95.16 ± 12.06	71.61 ± 8.27	<0.001	67.44 ± 8.47	<0.001	103.33 ± 15.98	74 ± 11.50	<0.001	75.48 ± 11.44	<0.001
BMI, kg/m^2^	35.51 ± 5.19	27.27 ± 3.81	<0.001	25.15 ± 3.43	<0.001	34.08 ± 4.83	24.49 ± 3.12	<0.001	24.85 ± 3.07	<0.001
WC, cm	111.46 ± 10.07	88(82.5,97)	<0.001	86(80,92.5)	<0.001	110 ± 9.25	89.89 ± 8.22	<0.001	86.75 ± 8.06	<0.001
VFA, cm^2^	143.25 ± 59.02	79.45 ± 22.08	0.004	61.34 ± 19.97	<0.001	178.31 ± 56.29	59.03 ± 15.46	<0.001	72.90 ± 46.35	<0.001
SFA, cm^2^	463.65 ± 135.41	260.8(197.25,309.2)	0.008	236.00 ± 66.71	<0.001	330.2 ± 129.31	132.74 ± 37.03	<0.001	161.80 ± 55.96	<0.001
PMA, cm^2^	22.53 ± 3.98	18.01 ± 3.64	0.008	17.91(15.31,21.23)	<0.001	37.31 ± 5.89	30.54 ± 5.46	0.014	33.07 ± 4.82	0.002
eBF%	37.01 ± 6.76	34.97 ± 5.15	<0.001	32.11 ± 4.89	<0.001	31.84 ± 5.47	20.42 ± 4.02	<0.001	20.77 ± 3.22	<0.001
eFFMI	20.53 ± 0.69	17.56 ± 1.12	<0.001	16.92 ± 1.08	<0.001	22.99 ± 1.55	19.39 ± 1.61	<0.001	19.61 ± 1.77	<0.001

BMI, body mass index; WC, waist circumference; VFA, visceral fat area; SFA, subcutaneous fat area; PMA, psoas muscle area; %eBF, estimated body fat percentage; eFFMI, estimated fat-free mass index.

Data are presented as means ± standard deviations, or medians (Q25, Q75).

P values indicate statistical significance compared with baseline in each sex group.

**Table 3:** Time × sex coefficients of body composition and bone mineral density from generalized linear mixed models.

	Time × Sex	p-value	Time^2^ × Sex	p-value
BMI	0.21	0.822	1.14	0.317
WC	3.35	0.291	1.20	0.634
VFA	-50.54	**0.032**	-23.49	0.166
SFA	28.60	0.478	58.25	0.150
PMA	0.07	0.941	-0.07	0.942
eBF%	0.14	0.900	1.27	0.361
eFFMI	-1.42	**<0.001**	-0.90	**0.018**
FN BMD	0.008	1.000	-0.003	1.000
TH BMD	-0.001	0.949	-0.03	**0.009**
LS BMD	-0.10	1.000	-0.09	1.000
P1NP	34.13	**0.003**	32.43	**0.001**
N-MID	6.80	1.000	10.57	1.000
β-CTX	329.65	**0.009**	285.74	**0.004**

BMD, bone mineral density; FN, femoral neck; TH, total hip; LS, lumbar spine; P1NP, procollagen type I amino-terminal propeptide; N-MID, N-terminal osteocalcin; β-CTX, β-cross-linked C-telopeptide of type I collagen.

Sex coefficients indicate men’s direction. Time indicates 6 months and Time^2^ indicates 12 months. The bold values indicat p-value < 0.05.

### 3.3 Changes in bone mineral density after sleeve gastrectomy

**Figure 1:**
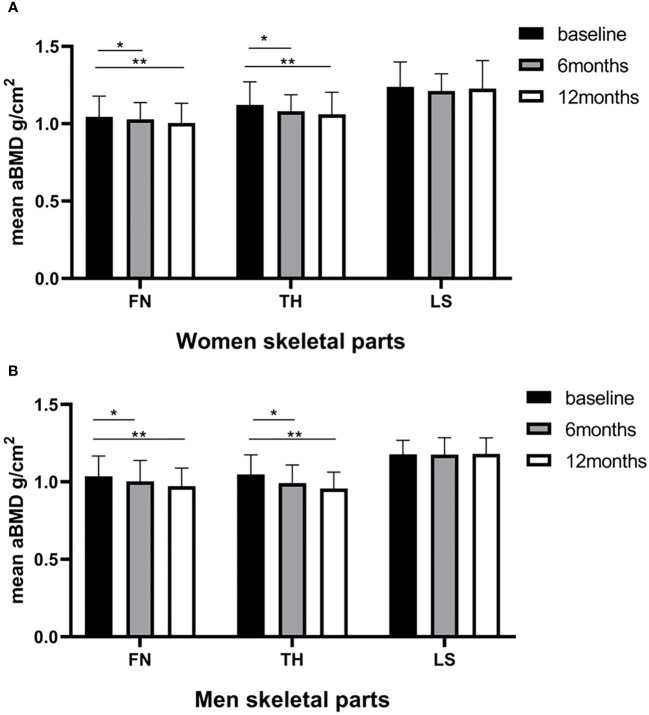
Baseline and postoperative bone mineral density (g/cm^2^) of the femoral neck (FN), total hip (TH), and lumbar spine (LS) according to the time of surgery. **(A)** women; **(B)** men. Statistical significance was calculated using a paired-samples t-test. *P < 0.05 versus baseline; **P < 0.001 versus baseline.

In our cohort, no cases of osteoporosis or fractures were reported, while clinically significant reductions in the BMD (> 0.03 g/cm^2^) at one or more sites (25) were observed in 36 patients (87.80%). The changes in bone mineral density after sleeve gastrectomy are shown in **Figure 1**. For women, the mean FN BMD (1.045 ± 0.133 vs. 1.004 ± 0.127 g/cm^2^; P < 0.001) and TH BMD (1.121 ± 0.149 vs. 1.060 ± 0.143 g/cm^2^; P < 0.001) decreased significantly across the 12 months. The same downward trend was observed in men. The mean FN BMD (1.036 ± 0.131 vs. 0.972 ± 0.117 g/cm^2^; P < 0.001) and TH BMD (1.049 ± 0.125 vs. 0.957 ± 0.107 g/cm^2^; P < 0.001) decreased significantly across the 12 months in men. The *post hoc* statistical power (%) of FN BMD loss within 6 months in the women group was 93.82%, and for the FN BMD loss within 12 months in the women and men group, it was 86.88% and 55.56% respectively. Sex-specific responses of SG revealed a more significant bone loss in men than in women at TH (a loss of BMD of 8.62% vs 5.44% across 12 months). The difference in FN BMD changes between women and men did not reach statistical significance (**Table 3**). However, LS BMD showed no changes at 6 months and 12 months postoperatively in both sexes (p > 0.05).

### 3.4 Changes in bone turnover markers after sleeve gastrectomy

**Figure 2:**
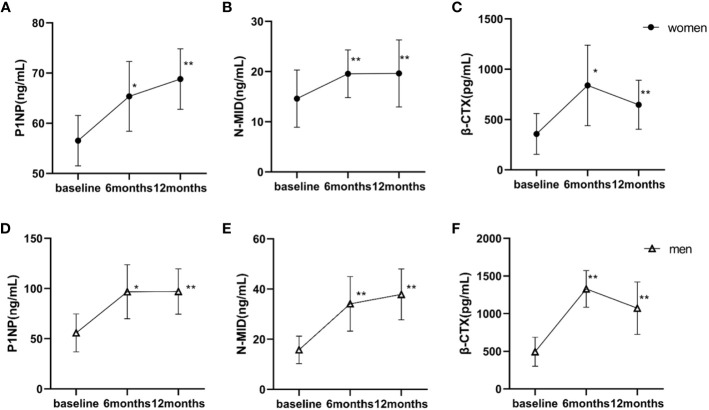
Baseline and postoperative bone turnover markers according to the time of surgery. Statistical significance was calculated using a paired-samples t-test. **(A–C)** women; **(D–F)** men. *P < 0.05 versus baseline; **P < 0.001 versus baseline. P1NP, procollagen type I amino-terminal propeptide; N-MID, N-terminal osteocalcin; β-CTX, β-cross-linked C-telopeptide of type I collagen.

The changes in measured bone turnover markers were shown in **Figure 2**. Compared with the baseline, all measured bone turnover markers increased for 12 months. In women, the P1NP and N-MID levels (56.54 ng/mL and 14.60 ng/mL, respectively) increased persistently to peak values at 12 months postoperatively (68.82 ng/mL and 19.63 ng/mL, respectively; both P < 0.001). The β-CTX levels continued to increase and reached its peak at 6 months (356.90 vs. 839.31 pg/mL; P < 0.05), remaining at a high level until 12 months (356.90 vs. 646.42 pg/mL; P < 0.001). Similarly in men, the P1NP and N-MID levels (55.79 ng/mL and 15.75 ng/mL, respectively) increased persistently to peak values at 12 months postoperatively (97.01 ng/mL and 37.88 ng/mL, respectively; both P < 0.001). The β-CTX levels continuously increased, reaching a peak at 6 months (493.67 vs. 1329.63 pg/mL; P < 0.001) and remaining at a high level until 12 months (493.67 vs. 1072.26 pg/mL, P < 0.001). The GLMMs confirmed higher levels of P1NP and β-CTX in men versus women at both 6 months and 12 months. The difference in N-MID level changes between women and men did not reach statistical significance (**Table 3**).

### 3.5 Correlation between body composition changes and reduction in bone mineral density

To detect potential body composition variables related to the BMD loss, correlation analysis and subsequent stepwise multiple linear regression analysis were performed in both sexes for 6 months and 12 months after SG separately. During the first 6 months post-SG, the FN BMD loss was positively associated with the percent reduction in eFFMI after adjusting age and %EWL in women (adjusted β = 0.77, P = 0.004, adjusted R^2 = ^0.54) (**Supplementary Table 3**), while the FN BMD loss was positively associated with the percent reduction in SFA in men (r = 0.93, P = 0.007) (**Supplementary Table 1**). For 12 months post-SG, the FN BMD loss was negatively associated with the percent reduction in VFA in both women and men. The significance remained after adjusting age and percentage change in waist circumference in women (adjusted β = -0.58, P = 0.027, adjusted R^2 = ^0.42) and adjusting age as well as percentage change in total triglycerides in men (adjusted β = -0.68, P = 0.032, adjusted R^2 = ^0.39) (**Supplementary Table 3**). For TH, TH BMD loss was positively associated with a percent reduction in waist circumference (r = 0.45, P = 0.028) in women (**Supplementary Table 2**). Sex-specific factors were found in different bone sites. Furthermore, changes in various body compositions are associated with FN BMD loss at different time points. This indicated that changes in body composition might play roles in BMD loss in a dynamic and nonlinear fashion.

## 4 Discussion

This is the first study to analyze the effects of changes in body composition measured by MRI on BMD loss at three different sites within 12 months after SG. As demonstrated in our study, BMD significantly decreased at 6 months after SG and continued to decline for as long as 12 months. The cumulative reduction occurred prominently in TH BMD, followed by FN BMD, with no decline in LS BMD in both sexes and men experienced greater loss in TH BMD compared to women. Additionally, our data indicated that the reduction in various body composition parameters may has different impacts on BMD loss in a site-specific manner, which shows the limitation of %TWL as a mediator in this process. This supports the necessity of delineating body composition in relevant studies.

Using MRI, a gold-standard imaging modality for accurately measuring body composition, our data demonstrated a possible protective effect of VFA loss on FN BMD 12 months post-SG in both women and men. This finding is similar to another Chinese study, wherein the VFA was measured using DXA (26). Our longitudinal perspective further solidifies the cross-sectional findings of the inverse association between VFA and BMD (27, 28). This association was not significant for TH BMD or LS BMD, which suggests that there might be a greater benefit from VFA reduction on cortical bone than trabecular bone after SG (29). The mechanism by which VFA affects BMD in our patients remains unclear. Along with the reduction in VFA, the visceral fat-secreted pro-inflammatory cytokines such as interleukin-6 and tumor necrosis factor alpha (30) which increase bone resorption and decrease bone formation partly explain the probable protective effects of VFA reduction in the process. Theoretically, the improvements in sex hormone levels (31, 32) or the balance of the growth hormone-insulin-like growth factor 1 axis (33) after SG may also contribute to this process.

It should be noted that reduction in adipose tissue documented the opposite effect on BMD loss. The SFA reduction exhibited a detrimental effect on the FN BMD loss 6 months post-SG in our longitudinal study, which is similar to the findings of other studies measuring the body composition using DEXA (11, 34). Furthermore, the decrease in waist circumference composed of VFA and SFA was positively associated with TH BMD loss in women at 12 months post-SG. This could be attributed to a decrease in the partial mechanical load, conversion of androgens to estrogens in adipose tissue, serum leptin levels, insulin growth factor, and insulin production which in turn stimulates bone formation (35). However, Wang et al. (36) reported SFA had no relation with BMD in Chinese women. In other cross-sectional studies, positive (37) and negative (9) relations between SFA and BMD were reported in Chinese people. These contradictory results reflect the complexity of the effects of fat mass on BMD. The opposite effect of fat mass on BMD underscores the importance of body composition subdivision in SG patients to further understand the pathophysiological mechanisms that influence BMD.

Lean body mass has been reported to be a protective factor for BMD. The positive correlation observed between eFFMI reduction and FN BMD loss in women for the first 6 months post-SG was in line with previous evidence (8, 9). Although lean mass reduction is an important component of %EWL, the correlation remained statistically significant even adjusting for %EWL in our study. This emphasized the specific effect of lean mass on BMD except for gravitational loading. Their biomechanical coupling and numerous shared endocrine and paracrine properties (38, 39) might explain the adaptations of bone to SG-related muscle reduction. However, the reduction in PMA was not associated with BMD loss in our results. Although PMA at L3 has been reported as an effective surrogate for overall muscle mass, the lack of local strain (40) and paracrine effects on remote FN and TH may account for the absence of a statistically significant association in our population.

Along with a continuous decrease in total body weight, the regional body composition parameters changed at different rates and magnitudes over 12 months after BS. As demonstrated in our study, regional body composition decreased more significantly during the first 6 months after SG. In additional to the mechanical unloading of body weight, the secretion and metabolic status of fat or muscle mass also seem to be involved in the pathophysiological mechanism of SG-related bone changes (41). Therefore, differences in the physiologic nature of the regional body composition parameters and their relationship with BMD might contribute to the site-specific features of BMD loss. Moreover, our results in both genders indicate different determinants of FN BMD loss at 6 months and 12 months after SG. The asynchronous decrease in the body weight and BMD, along with their nonlinear interaction may account for these dynamic discrepancies.

Our findings suggest that women patients should monitor changes in lean mass, as those with significant loss of eFFMI may experience a substantial reduction in FN BMD. Therefore, it is recommended that these patients incorporate interventions such as strength training and adequate protein intake to prevent further decline in BMD. Additionally, our results indicate a positive association between SFA reduction and BMD loss, as well as a negative association between VFA reduction and BMD loss. A set of studies have demonstrated that increased visceral adiposity is linked to worse insulin resistance and an increased risk of cardiometabolic disease (42), while the preferential expansion of subcutaneous adipose tissue is associated with a more favorable metabolic profile (43). Hence, it is crucial for doctors to consider the distribution of adipose tissue rather than solely focusing on total fat reduction. Identifying patients who are prone to loss SFA after SG is crucial for early detection of individuals with a high risk of BMD loss.

However, this study has some limitations. Further studies with larger sample size are needed to validate our findings. A longer study period is required to explore the long-term BMD changes and even fractures occurrences. The quality of our study would be better if there were recorded data on patients’ activity level or nutritional intake which may affect bone health. This observational study can only propose the possible association between the body composition and BMD loss related to SG and causality cannot be assumed.

## 5 Conclusion

The FN and TH BMD decreased continuously over 12 months after SG in both sexes, with a greater reduction in TH BMD observed in men. Our results suggest that VFA and SFA may have opposite effects on BMD loss and eFFMI has a potential protective effect on BMD in women. Considering the disparate role of body composition changes in BMD loss at different bone sites and time points, accurately distinguishing between regional body composition from total body weight is crucial for analyzing the pathophysiological factors contributing to SG-related BMD loss.

## Data availability statement

The raw data supporting the conclusions of this article will be made available by the authors, without undue reservation.

## Ethics statement

The studies involving humans were approved by the Ethics Committee for Human Investigation of Shanghai Jiao Tong University School of Medicine affiliated Sixth People’s Hospital. The studies were conducted in accordance with the local legislation and institutional requirements. The participants provided their written informed consent to participate in this study.

## Author contributions

DY, JH, and YY designed the study. DY and RX analyzed the data, prepared the figures, and drafted the manuscript. YT and YX recruited participants and collected the data. HZ, WL, and PZ were responsible for bariatric surgery. YB, JH, YY, and HY contributed to the discussion and revised the manuscript. All authors contributed to the article and approved the submitted version.
