# Supplementary material for: Effect of regional body composition changes on bone density remodeling after sleeve gastrectomy
Source: Front Endocrinol (Lausanne). 2023 Sep 11;14:1238060. doi: 10.3389/fendo.2023.1238060 (PMC10518401; doi:10.3389/fendo.2023.1238060)
Supplement: Supplementary file 1 [file Table_1.docx]

**Supplementary Table 1** Correlations between changes in metabolic characteristics, body composition and BMD loss for women and men at 6 months after SG.

|  | Women | | | |  | Men | | | |
| --- | --- | --- | --- | --- | --- | --- | --- | --- | --- |
|  | FN BMD | | TH BMD | |  | FN BMD | | TH BMD | |
| % change | r | P value | r | P value |  | r | P value | r | P value |
| BMI | -0.098 | 0.751 | 0.018 | 0.953 |  | 0.515 | 0.191 | 0.054 | 0.900 |
| WC | 0.130 | 0.687 | 0.049 | 0.879 |  | 0.420 | 0.300 | 0.044 | 0.917 |
| VFA | -0.646 | 0.084 | 0.106 | 0.803 |  | -0.128 | 0.809 | 0.328 | 0.525 |
| SFA | -0.556 | 0.153 | -0.289 | 0.488 |  | 0.931 | **0.007** | -0.580 | 0.228 |
| PMA | -0.457 | 0.255 | -0.415 | 0.307 |  | 0.360 | 0.483 | -0.657 | 0.156 |
| %eBF | -0.103 | 0.739 | -0.014 | 0.963 |  | 0.569 | 0.141 | 0.070 | 0.869 |
| eFFMI | 0.681 | **0.010** | -0.111 | 0.718 |  | 0.296 | 0.477 | -0.425 | 0.294 |
| %EWL | 0.670 | **0.012** | 0.028 | 0.927 |  | -0.113 | 0.773 | -0.323 | 0.396 |
| TC | 0.146 | 0.650 | 0.062 | 0.849 |  | -0.095 | 0.822 | 0.276 | 0.508 |
| TG | 0.304 | 0.364 | 0.120 | 0.725 |  | -0.137 | 0.746 | 0.367 | 0.372 |
| HDL-C | -0.156 | 0.629 | 0.293 | 0.355 |  | 0.012 | 0.978 | 0.338 | 0.412 |
| LDL-C | 0.295 | 0.353 | -0.074 | 0.819 |  | -0.214 | 0.611 | 0.274 | 0.511 |
| FPG | -0.008 | 0.978 | 0.457 | 0.116 |  | -0.483 | 0.272 | 0.004 | 0.994 |
| HbA1c | 0.122 | 0.705 | 0.345 | 0.271 |  | -0.512 | 0.194 | -0.084 | 0.844 |
| FINS | 0.018 | 0.953 | -0.351 | 0.239 |  | 0.538 | 0.213 | 0.253 | 0.585 |
| HOMA-IR | 0.136 | 0.659 | 0.019 | 0.952 |  | 0.499 | 0.254 | 0.289 | 0.530 |
| HOMA-β | 0.141 | 0.646 | -0.039 | 0.900 |  | 0.416 | 0.354 | 0.069 | 0.883 |
| TSP | 0.275 | 0.441 | 0.369 | 0.263 |  | 0.318 | 0.442 | -0.305 | 0.557 |
| SA | 0.261 | 0.466 | 0.393 | 0.232 |  | 0.395 | 0.333 | -0.497 | 0.315 |

*Abbreviations*: *BMD* bone mineral density; *FN* femoral neck; *TH* total hip; *BMI* body mass index; *WC* waist circumference; *VFA* visceral fat area; *SFA* subcutaneous fat area; *PMA* psoas muscle area; *%eBF* estimated body fat percentage; *eFFMI* estimated fat-free mass index; *%EWL* excess weight loss; *TC* total cholesterol; *TG* total triglycerides; *HDL-C* high-density lipoprotein cholesterol; *LDL-C* low-density lipoprotein cholesterol; *FPG* fasting plasma glucose; *HbA1c* glycated hemoglobin; *FINS*, fasting insulin; *HOMA-IR* homeostasis model assessment of insulin resistance; *HOMA-β* homeostasis model assessment of β-cell function; *TSP* total serum protein; *SA* serum albumin.

**Supplementary Table 2** Correlations between changes in metabolic characteristics, body composition and BMD loss for women and men at 12 months after SG.

|  | Women | | | |  | Men | | | |
| --- | --- | --- | --- | --- | --- | --- | --- | --- | --- |
|  | FN BMD | | TH BMD | |  | FN BMD | | TH BMD | |
| % change | r | P value | r | P value |  | r | P value | r | P value |
| BMI | -0.037 | 0.862 | 0.213 | 0.307 |  | -0.133 | 0.681 | 0.253 | 0.428 |
| WC | 0.393 | 0.057 | 0.448 | **0.028** |  | -0.186 | 0.562 | 0.202 | 0.529 |
| VFA | -0.454 | **0.023** | -0.237 | 0.254 |  | -0.677 | **0.016** | -0.204 | 0.524 |
| SFA | 0.012 | 0.956 | 0.196 | 0.348 |  | -0.288 | 0.364 | 0.038 | 0.907 |
| PMA | 0.160 | 0.445 | 0.211 | 0.311 |  | -0.035 | 0.914 | 0.224 | 0.484 |
| %eBF | -0.019 | 0.930 | 0.232 | 0.264 |  | -0.119 | 0.713 | 0.289 | 0.363 |
| eFFMI | 0.085 | 0.686 | 0.098 | 0.643 |  | -0.189 | 0.556 | 0.234 | 0.464 |
| %EWL | 0.134 | 0.532 | 0.229 | 0.282 |  | -0.139 | 0.666 | 0.065 | 0.840 |
| TC | 0.308 | 0.187 | 0.346 | 0.135 |  | 0.042 | 0.903 | 0.121 | 0.722 |
| TG | 0.331 | 0.166 | 0.269 | 0.266 |  | -0.563 | 0.071 | -0.213 | 0.529 |
| HDL-C | -0.352 | 0.128 | -0.060 | 0.800 |  | 0.318 | 0.314 | 0.143 | 0.657 |
| LDL-C | 0.313 | 0.179 | 0.130 | 0.584 |  | 0.295 | 0.379 | 0.349 | 0.292 |
| FPG | -0.086 | 0.727 | 0.216 | 0.374 |  | -0.009 | 0.981 | -0.506 | 0.135 |
| HbA1c | 0.129 | 0.587 | 0.504 | **0.023** |  | -0.309 | 0.355 | -0.647 | **0.031** |
| FINS | -0.229 | 0.331 | -0.300 | 0.198 |  | 0.130 | 0.720 | 0.313 | 0.378 |
| HOMA-IR | -0.161 | 0.498 | -0.125 | 0.599 |  | 0.060 | 0.862 | 0.296 | 0.377 |
| HOMA-β | -0.047 | 0.850 | -0.389 | 0.100 |  | -0.002 | 0.995 | 0.313 | 0.348 |
| TSP | 0.031 | 0.893 | 0.017 | 0.942 |  | 0.392 | 0.207 | 0.617 | **0.033** |
| SA | 0.012 | 0.958 | -0.026 | 0.909 |  | 0.397 | 0.201 | 0.752 | **0.005** |

**Supplementary Table 3** Multiple stepwise linear regression analysis about the influencing factors independently associated with FN BMD loss during 12 months after SG.

|  |  | Women | | | |  | Men | | | |
| --- | --- | --- | --- | --- | --- | --- | --- | --- | --- | --- |
| Time post SG | Dependent variable | Independent variable | Adjusted β | P | Adjusted R2 |  | Independent variable | Adjusted β | P | Adjusted R2 |
| 6 months | ∆ FN BMD | ∆ eFFMI | 0.77 | 0.004 | 0.54 |  | - | - | - | - |
| 12 months | ∆ FN BMD | ∆VFA | -0.58 | 0.027 | 0.42 |  | ∆VFA | -0.68 | 0.032 | 0.39 |

∆FN BMD in women at 6 months post SG adjusted for age and %EWL.

∆FN BMD in women at 12 months post SG adjusted for age and percent change in waist circumference.

∆FN BMD in men at 12 months post SG adjusted for age and percent change in total triglycerides.
